# Design and testing of a 96-channel neural interface module for the Networked Neuroprosthesis system

**DOI:** 10.1186/s42234-019-0019-x

**Published:** 2019-02-15

**Authors:** Autumn J. Bullard, Samuel R. Nason, Zachary T. Irwin, Chrono S. Nu, Brian Smith, Alex Campean, P. Hunter Peckham, Kevin L. Kilgore, Matthew S. Willsey, Parag G. Patil, Cynthia A. Chestek

**Affiliations:** 1 Department of Biomedical Engineering, University of Michigan, Ann Arbor, MI USA; 2 Department of Biomedical Engineering, Case Western Reserve University, Cleveland, OH USA; 3 Department of Orthopaedics, MetroHealth Medical Center, Cleveland, OH USA; 4 Research Service, Louis Stokes Cleveland Department of Veterans Affairs Medical Center, Cleveland, OH USA; 5 Department of Neurosurgery, University of Michigan, Ann Arbor, MI USA; 6 Department of Neurology, University of Michigan, Ann Arbor, MI USA; 7 Department of Anesthesiology, University of Michigan, Ann Arbor, MI USA; 8 Department of Electrical Engineering and Computer Science, University of Michigan, Ann Arbor, MI USA

**Keywords:** Brain machine interface, Functional electrical stimulation, Implantable, Neural interface, Low power

## Abstract

**Background:**

The loss of motor functions resulting from spinal cord injury can have devastating implications on the quality of one’s life. Functional electrical stimulation has been used to help restore mobility, however, current functional electrical stimulation (FES) systems require residual movements to control stimulation patterns, which may be unintuitive and not useful for individuals with higher level cervical injuries. Brain machine interfaces (BMI) offer a promising approach for controlling such systems; however, they currently still require transcutaneous leads connecting indwelling electrodes to external recording devices. While several wireless BMI systems have been designed, high signal bandwidth requirements limit clinical translation. Case Western Reserve University has developed an implantable, modular FES system, the Networked Neuroprosthesis (NNP), to perform combinations of myoelectric recording and neural stimulation for controlling motor functions. However, currently the existing module capabilities are not sufficient for intracortical recordings.

**Methods:**

Here we designed and tested a 1 × 4 cm, 96-channel neural recording module prototype to fit within the specifications to mate with the NNP. The neural recording module extracts power between 0.3–1 kHz, instead of transmitting the raw, high bandwidth neural data to decrease power requirements.

**Results:**

The module consumed 33.6 mW while sampling 96 channels at approximately 2 kSps. We also investigated the relationship between average spiking band power and neural spike rate, which produced a maximum correlation of R = 0.8656 (Monkey N) and R = 0.8023 (Monkey W).

**Conclusion:**

Our experimental results show that we can record and transmit 96 channels at 2ksps within the power restrictions of the NNP system and successfully communicate over the NNP network. We believe this device can be used as an extension to the NNP to produce a clinically viable, fully implantable, intracortically-controlled FES system and advance the field of bioelectronic medicine.

## Background

According to the National Spinal Cord Injury Statistical Center, there are approximately 282,000 people living with chronic spinal cord injury (SCI), with 17,000 new cases occurring each year (National Spinal Cord Injury Statistical Center 2016). Damage to the spinal cord can disrupt the pathway of signals sent between the brain and the body and may result in partial or complete loss of both motor and sensory functions below the level of injury. The loss of these functions can have a major impact and severely interfere with activities of daily living related to arm and hand function, walking, bladder and bowel control, and trunk stability. Interestingly, restoration of arm and hand function was ranked as the highest priority amongst individuals with tetraplegia and could significantly improve quality of life (Anderson 2004). Functional electrical stimulation (FES), a technique that uses pulses of electrical current to generate contractions of muscles, has been beneficial in assisting and improving the impaired motor function in individuals with SCI. FES has made great strides in improving not only hand function (Peckham et al. 1980; Alon and McBride 2003; Popovic et al. 2002; Kilgore et al. 2008), but also walking (Daly et al. 2011; Thrasher et al. 2006), and controlling bladder functions (Gaunt and Prochazka 2006).

FES systems have been controlled by the user via physical switches, shoulder motion, and wrist position, which allows patients to cycle through pre-programmed stimulation patterns (Prochazka et al. 1997; Snoek et al. 2000; Handa et al. 1992; Johnson et al. 1999; Smith et al. 1987). These previous methods provided somewhat coarse and binary control of the limb. Current FES systems use residual myoelectric activity or joint angles as a method of control to achieve more finely tuned movements (Memberg et al. 2014; Smith et al. 1987). Although these current methods may work for individuals with partial paralysis, they can be unintuitive for patients and only provide a few degrees of freedom. Further, in high cervical SCI, there is little or no residual motor function to provide an appropriate input stimulus to control such systems. Thus, a control solution which can provide more function to patients with all levels of injury is needed.

Brain machine interfaces (BMIs) have demonstrated great potential for generating control signals for prosthetic devices (Chapin et al. 1999; Collinger et al. 2013; Gilja et al. 2015; Hochberg et al. 2012; Velliste et al. 2008). The capability of BMIs to decode intended movement directly from the brain can be used to control FES systems, potentially restoring natural function to patients with all levels of spinal cord injury. Recently, groups have successfully used intracortically recorded signals from Utah microelectrode arrays (Blackrock Microsystems, Salt Lake City, UT) to predict movements or electromyogram (EMG) patterns to control FES systems (Ajiboye et al. 2012; Bouton et al. 2016; Ethier et al. 2012). Bouton et al. demonstrated that multiunit activity in a paralyzed human could be used to control muscle activation directly and provide continuous control of isolated finger movements and six different wrist and hand postures (Bouton et al. 2016). Most recently, Ajiboye et al. enabled a person with a C3 level injury to perform self-feeding activities using Utah array-controlled FES (Ajiboye et al. 2017). Although cortical control of FES is promising, current BMIs still require percutaneous leads connecting indwelling electrodes to external recording devices that can increase the risk of infection and limit portability. Therefore, wireless technology is required to move towards a fully implantable and clinically viable device.

Many groups have designed and built custom implantable, wireless neural recording devices (Aziz et al. n.d.; Gao et al. 2012; Moo Sung Chae et al. 2009; Park et al. 2018; Wattanapanitch and Sarpeshkar 2011). However, none have been FDA approved or tested in humans. One of the major challenges in translating these systems into clinical use is the high bandwidth needed to access individual neural waveforms. The need to acquire, process, and transmit this broadband neural data dramatically increases the power requirements of the device, which results in large batteries with low battery life. For example, Borton et al. developed a 100 channel, hermetically sealed, implantable neural recording system. This device transmits broadband data at 24Mbps, requires 90.6 mW, and can last 7 h on a medical grade 200mAh battery (Borton et al. 2013). Similarly, Miranda et al. developed a 32-channel system of primarily off the shelf components that delivered broadband data at 24 Mbps, using 142 mW (Miranda et al. 2010). This system can last up to 33 h but requires two 1200 mAh batteries. In all cases, the high power requirement prevents the use of compact batteries with adequate battery life practical for an implantable device.

One method for saving power is to reduce the system bandwidth by focusing on relevant BMI features of the intracortical signals. Intracortical BMIs typically analyze the action potential or “spike” frequencies. Spikes are detected in the broadband signal by setting thresholds and counting the number of crossings in regular time intervals. These spike counts can be used to predict both continuous and discrete movements. Spike sorting individual neurons may be beneficial if an electrode is recording neurons with independent tuning patterns, otherwise, if electrodes primarily have only one neuron or similarly tuned neurons then a substantial performance gain is not expected. Numerous studies have used thresholded spikes in BMI experiments and have shown that there is minimal or no performance loss when compared to sorted action potentials extracted from the broadband data (Chestek et al. 2011; Fraser et al. 2009). In investigation of spike sorted data in comparison to thresholded data for the use in BMIs we have previously shown that spike sorting does not substantially improve decoding performance. Using a Naïve Bayes classifier with both thresholded and spike sorted data, we demonstrated percent accuracy only changed by an average of 5% and the correlation coefficient only differed by 0.015 (Christie et al. 2015). Thus, instead of transmitting the entire broadband signal, only the spike counts are needed to generate commands (Harrison et al. 2009). This immensely compresses the data, decreasing the required data rate for transmission. Beyond using spike counts, Stark and Abeles found that most of the decoding information received from spikes could also be found in the signal frequency band of 300–6000 Hz, which includes the spike waveform frequencies (Stark and Abeles 2007). We have previously shown this bandwidth can be further reduced to 300–1000 Hz and extract similar intracortical information (Irwin et al. 2016). We refer to this 300–1000 Hz band as the “spiking band”. Specifically, we developed a 16-channel wireless neural interface to assess the power savings and BMI decoder performance of this approach. We compared the decode performance of continuous finger position using a Wiener filter between high bandwidth data using spike counts and low bandwidth data using the spiking band. That study showed that instead of transmitting the entire broadband signal, extracting only signal power within a narrow frequency band allowed for a reduced sampling rate and resulted in a power savings of roughly 90% with only a 5% performance loss of the accuracy of the decoder (Irwin et al. 2016). This approach could be applied in the context of existing fully implantable systems to decrease power consumption and allow for practical neural control of FES devices.

One efficient way to move the field towards a fully implantable intracortical BMI-FES system for clinical use may be to merge a neural recording device with an already existing fully implantable FES system. Using similar signal processing techniques from our previous wireless device (Irwin et al. 2016), we developed a novel 96-channel intracortical recording device to be used as an extension to the modular, fully implantable FES system developed at Case Western Reserve University. In this paper we describe a feasibility study for power, performance, and form factor to mate with their system and the prospect of an implantable cortical-controlled FES system.

## Methods

### Networked Neuroprosthesis system

The Networked Neuroprosthesis (NNP) is a system of implantable modules used to record residual EMG and perform many combinations of neural stimulation for controlling grasp and other motor functions (Smith et al. 2005). While other implantable FES systems and modular networks have been developed (Ghoreishizadeh et al. 2017; Guiraud et al. 2014; Jovičić et al. 2012), to our knowledge, the NNP is currently the only fully implantable FES system in initial human testing for hand function. It consists of a single central power module, and multiple actuator and sensor modules that are all interconnected via a network cable. The central power module is used for battery housing and wireless transfer. It manages power distribution and has the capability to monitor and program functions to other modules throughout the network. The actuator module is responsible for providing stimulation to muscles or nerves while the sensor module records biopotential data. The network cable provides the power and high-speed data link for the modules with a two wire Controller Area Network-like (CAN) bus protocol (Kilgore et al. 2007; Smith et al. 2005).

The current NNP sensor modules are designed with two bipolar channels to record EMG. These sensor modules are not equipped to support 96-channel intracortical neural recordings. However, the NNP system architecture was designed as a platform technology to accept new modules with added functionality. Theoretically, a separate 96-channel neural recording module can be added to the NNP system to facilitate cortical controlled FES. This module would need to adhere to the guidelines of the standard NNP sensor modules: fit into the approved 1 cm × 3 cm hermetically sealed packaging of the sensor modules, include CAN capabilities, and meet the requirements of the 50 kbps network bandwidth and the target power consumption of about 30 mW. Because the NNP has been cleared for human testing and offers an open architecture, we chose this as our base system to test the feasibility of a fully implantable, cortically controlled FES system. The final system will record neural data from a 96-channel Utah array and generate command signals for grasping using low power circuitry and the spiking band feature extraction technique. Herein, we present the design and experimental results of this neural recording module prototype, including validation using novel datasets with intracortical recordings from rhesus macaques performing finger movements.

### Cortical controlled FES system overview

**Fig. 1:**
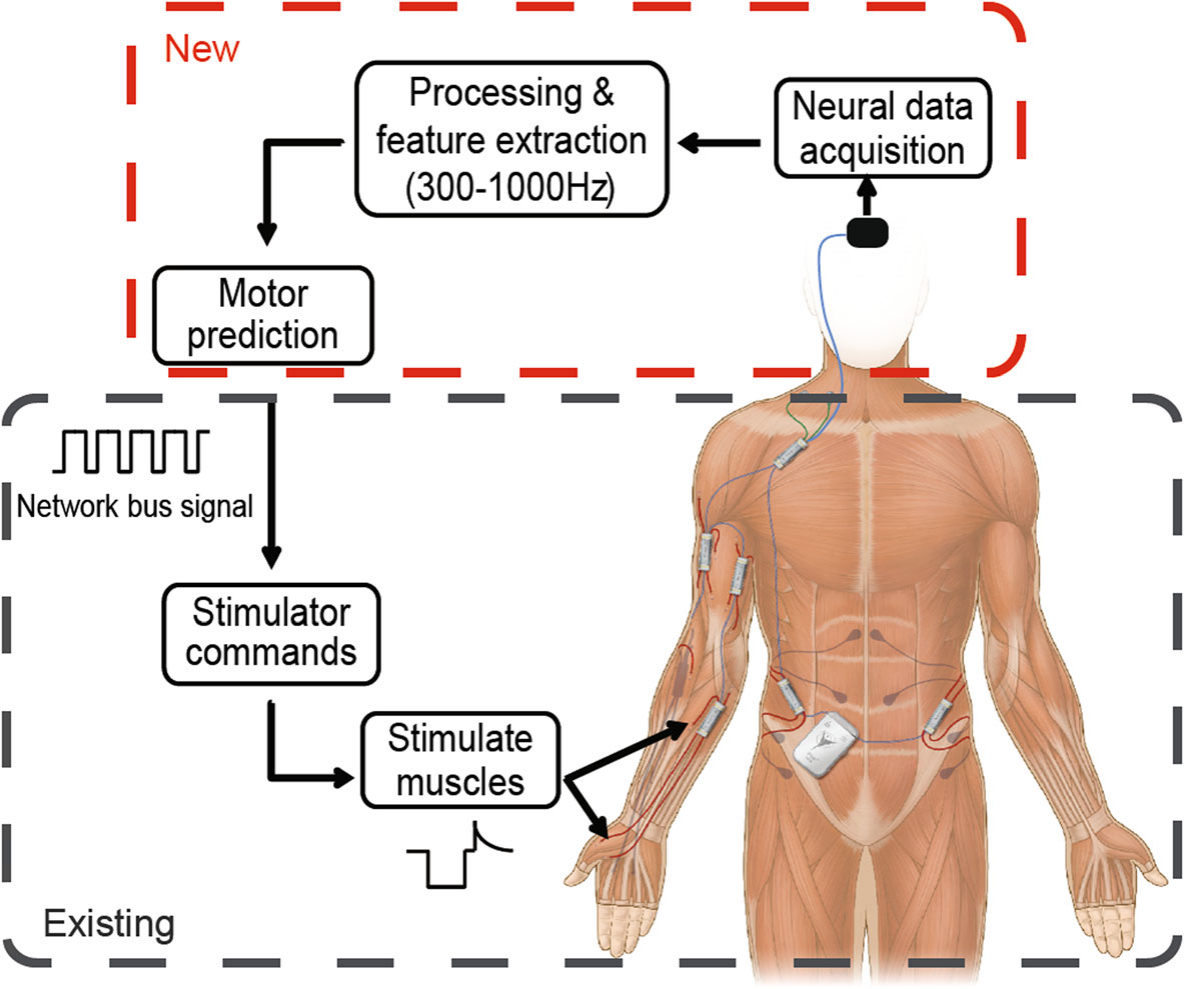
Concept diagram for brain-controlled FES. The grey box denotes the existing NNP system and the red box denotes the planned novel module

The envisioned intracortical FES system is shown in Fig. 1. The existing NNP architecture is used for muscle stimulation, power, and communication, while the novel intracortical recording module presented here generates control signals based on user intention. The novel recording module records data from a 96-channel Utah array in motor cortex, extracts the signal power in the 300–1000 Hz frequency band, and will ultimately predict the user’s motor intention using decoding techniques similarly to (Irwin et al. 2016). That intention is then converted into appropriate stimulation patterns by the power module and stimulates the paralyzed limb via the actuator module. The central power module of the NNP provides power to all sensor and actuator modules and provides a communication pathway both between modules and for external programming.

The Utah array will be directly wire bonded to the neural recording module in a hermetically sealed capsule which will be secured to the skull, similar to what is seen with the Responsive Neurostimulator system (Neuropace). Securing this module to the skull should limit cable length and the possibility of excessive noise or interferance of the signal. The neural recording module will then be connected to other modules of the NNP throughout the body via the network cable.

### Recording module hardware design

**Fig. 2:**
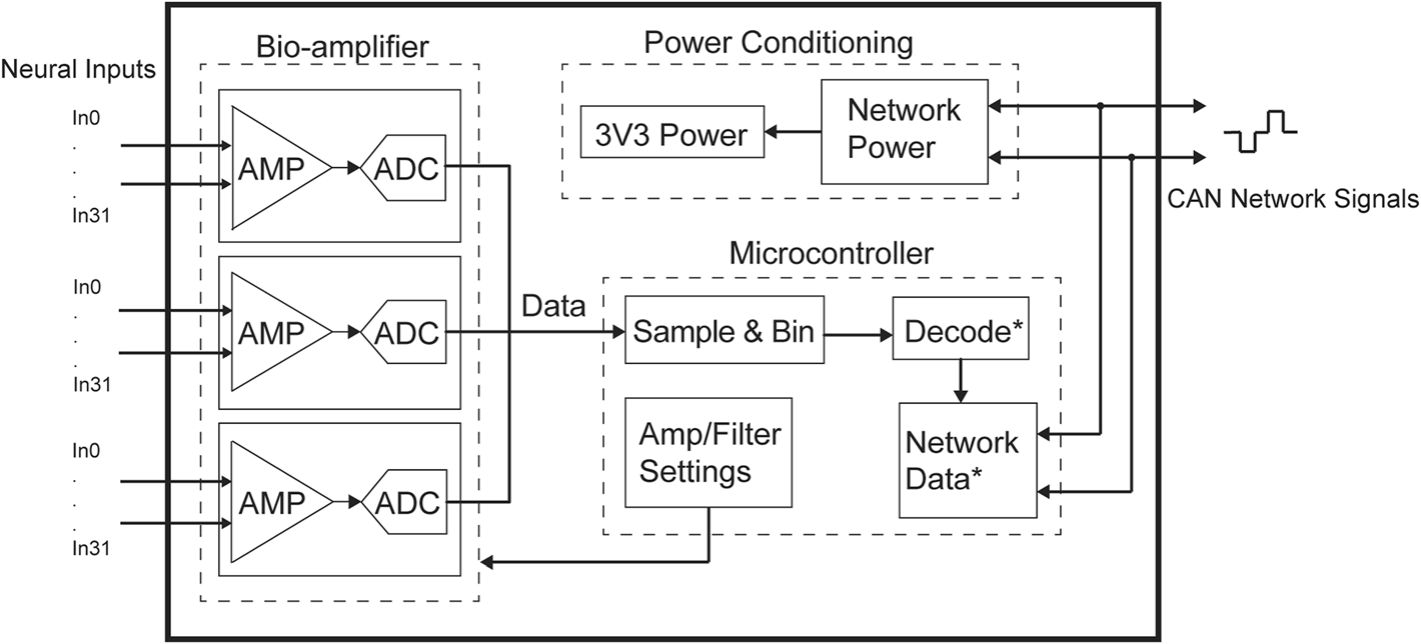
Block diagram of the neural recording module. (*) indicates future work not presented here

The initial design specifications for this module, summarized in Table 1, were determined based on the existing design of the Networked Neuroprosthesis from Case Western and from the design of our previous wireless system (Irwin et al. 2016). The recording module is described by the block diagram in Fig. 2. It has a 96-channel front-end which filters and digitizes the incoming neural data. The absolute value of the data on each channel is binned by the central microcontroller of the device by averaging over a given time interval to calculate the mean signal power. Decoding and communication over the CAN bus have not yet been implemented. However, in the future, the power on all channels will be decoded by the central microcontroller to predict the user’s intended grasp type. Finally, the decoded grasp type will be sent over the network to the NNP modules for appropriate muscle stimulation. Here, we validate the module’s ability to record the neural data at power levels that will work in conjunction with the entire system.

**Table 1:** Neural Recording Module Design Specifications

Parameter	Value
Area	1.0 × 4.0 cm
No. channels	96^*^
ADC resolution	16 bits
Amplifier input noise	2.4 μV_RMS_
CPU clock	8 MHz
Low-pass filter	0.1–20 kHz^*^
High-pass filter	0.1–500 Hz^*^
Sampling rate	2.17 kSps (96 channels)
	32.05 kSps (1 channel)
Supply voltage	3.3 V

^*^Configurable in software

The module was implemented using entirely commercial, off-the-shelf components. The front-end consists of three Intan RHD2132 32-channel bioamplifiers that combine analog and digital filters and a multiplexed 16-bit ADC. The upper and lower bandwidths of the amplifiers and number of active channels can be easily configured by the central microcontroller (MCU) via a standard four-wire SPI interface. The MCU has three USART ports that are configured for SPI to transmit data from all three amplifiers simultaneously. The lower cutoff frequency of the amplifier bank is adjustable from 0.1–500 Hz, while the upper cutoff range is 100–20,000 Hz. The ADC sampling rate is controlled by the MCU, which was set to approximately 2 kSps per channel when using all 96 channels to measure signal power, or approximately 30 kSps for a single channel’s single unit recording. The power consumption of the amplifiers is proportional to the upper cut off frequency and scales at 7.6 μA/kHz per channel. The chosen upper cut off frequency is the major factor in the power consumption of the amplifiers. The upper cut off frequency also determines the minimum sampling rate that can be applied, which is a major factor on the MCU processing and power consumption of the overall system.

A 32-bit Atmel ATUC3C2256C MCU serves as the central controller and data processor, configuring the front-end amplifiers, controlling the rate of data flow, as well as eventually communicating with existing NNP circuitry via a CAN-like bus. Feature extraction from the data is performed on the MCU by averaging the absolute value of the data over a specified bin size that is programmed, which we define as spiking band power. The MCU is clocked via an external crystal oscillator at 8 MHz which is internally divided for device operation and SPI communication. The MCU is programmed from an external computer via a ten-pin AVR JTAG interface, and system configuration settings can be easily modified in the application code.

The MCU’s Direct Memory Access (DMA) controller allows sampling of the bioamplifiers without processor oversight and was used to save power. The system is configured to initialize the 8 MHz clock, USART modules, and Intan amplifiers using the fully active MCU at startup. After initialization, the DMA controller is then set up to sample data using the USART peripheral (in SPI mode) and transfer it to internal memory. Once it is enabled, the MCU enters a low-power sleep mode. Since the USART module has a lower bit resolution than the Intan amplifiers, a small amount of glue logic was required to drive the MCU’s SPI interface to each amplifier while the MCU remained asleep. A D-flip flop, AND-gate, and necessary propagation delay circuitry were added to each chip select line on each SPI bus to drive the MCU’s SPI interface for compatibility with each amplifier while the MCU remained asleep.

The remaining circuitry is responsible for network communication and power conditioning. This section uses identical components and a similar layout as the existing modules of the NNP. The network is designed to be DC isolated and is used to communicate with other NNP modules using the CAN bus protocol. The power conditioning circuitry will ultimately be responsible for harvesting power from the network.

### Printed circuit board layout

NNP remote modules are designed to have several 1 cm wide rigid printed circuit board (PCB) panels, connected via flexible PCBs. This enables the modules to be folded to fit in a 1 cm wide enclosure, while maintaining component layout space. These modules can additionally accommodate variable length enclosures, and designers have the choice of trading off module length for circuit board density and complexity.

**Fig. 3:**
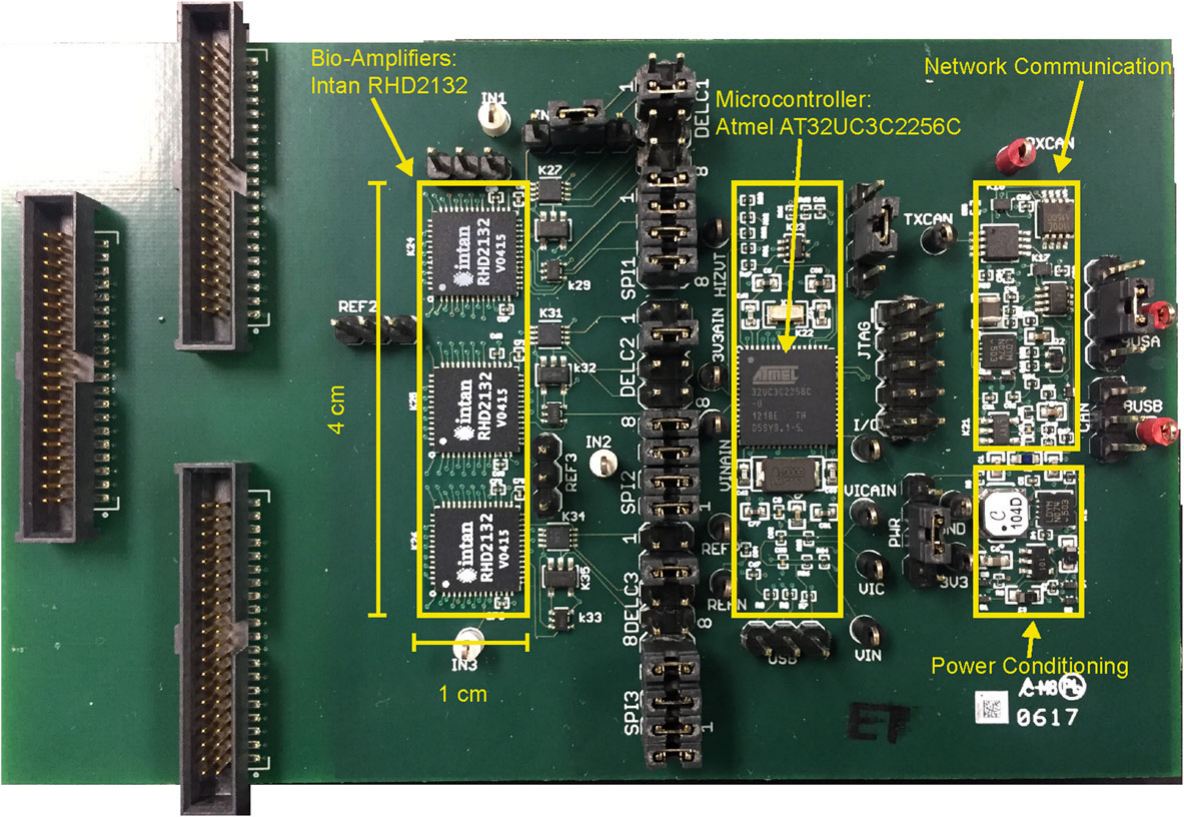
Prototype board used for testing. Three panels (left to right): bioamplifiers, microcontroller, network and power circuitry

**Fig. 4:**
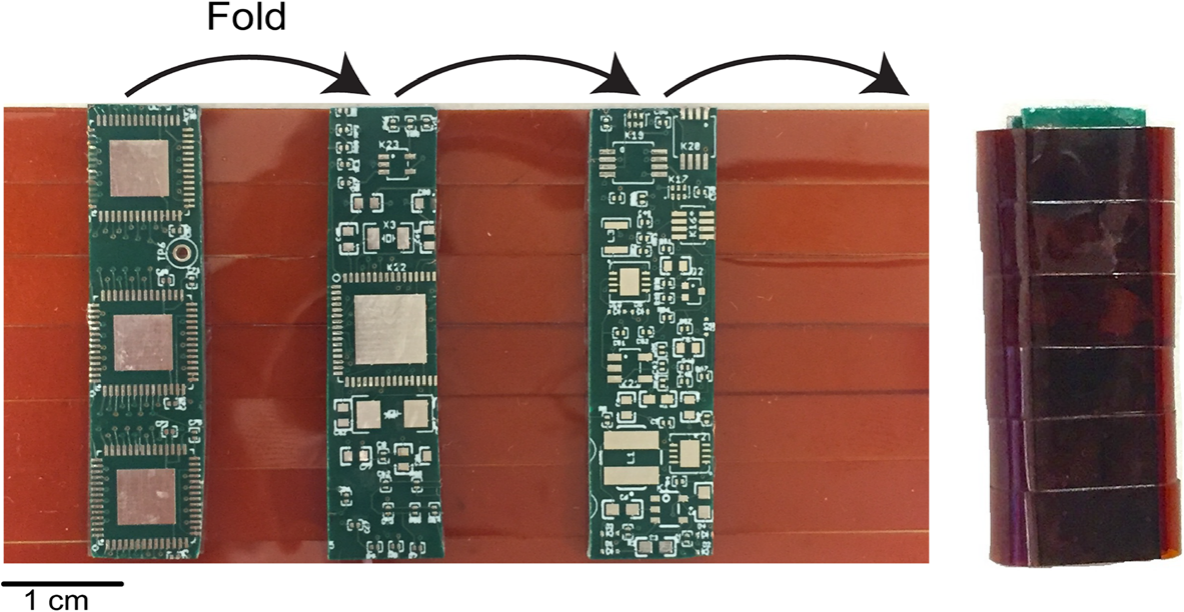
(Left) Example mockup of final design with flexible circuitry. (Right) Demonstration of folded board to fit within the NNP module canister

The novel module described thus far was prototyped on a six-layer printed circuit board. The prototype layout is shown in Fig. 3. All active circuitry fit within three 1 cm × 4 cm panels in order to test the feasibility of fitting within an NNP package. Ultimately, a fourth panel will be used comprised of all bond pads to connect internal circuitry to the external electrode array. The limiting factor was the 32-channel Intan chips. All three Intan bioamplifiers were put on one panel with signal lines extending out from the side of the board. This solution required an extension of the board length from 3 cm for a typical NNP module to 4 cm for the novel module. A total of 12 signal lines pass between the bioamplifier and microcontroller panels, four SPI lines for each amplifier, all on one inner layer. In addition, only four lines pass between the microcontroller panel and the communication and power panel. This will enable future versions to be fabricated with a rigid-flex circuit board that allows folding to fit within an NNP remote module enclosure (illustrated in Fig. 4). Most of the components outside of these panels are only used to facilitate benchtop testing and are not required for device operation. This includes test points, programming headers, and Samtec connectors that can connect directly to a Utah array head stage (Blackrock Microsystems). The remaining glue logic components were later added unconstrained to the outlined panels to avoid any major design changes; however, they could fit with the necessary adjustments.

### Experimental design and device validation

We explored the module’s ability to record in single channel (30 kSps) and multichannel (2 kSps) modalities. The sampling rate is approximate and can vary slightly based on code configurations. In each modality, the power consumption and its correlation to channel count was analyzed. In addition, we used the module to investigate the relationship between spiking band power and the firing rate of action potentials. This neural recording module was validated using pre-recorded data that was played back through the module and in vivo intracortical recordings directly from a rhesus macaque. All animal procedures were approved by the University of Michigan Institutional Review Board and the Institutional Animal Care & Use Committee.

#### Surgical Implantation & Electrophysiology

**Fig. 5:**
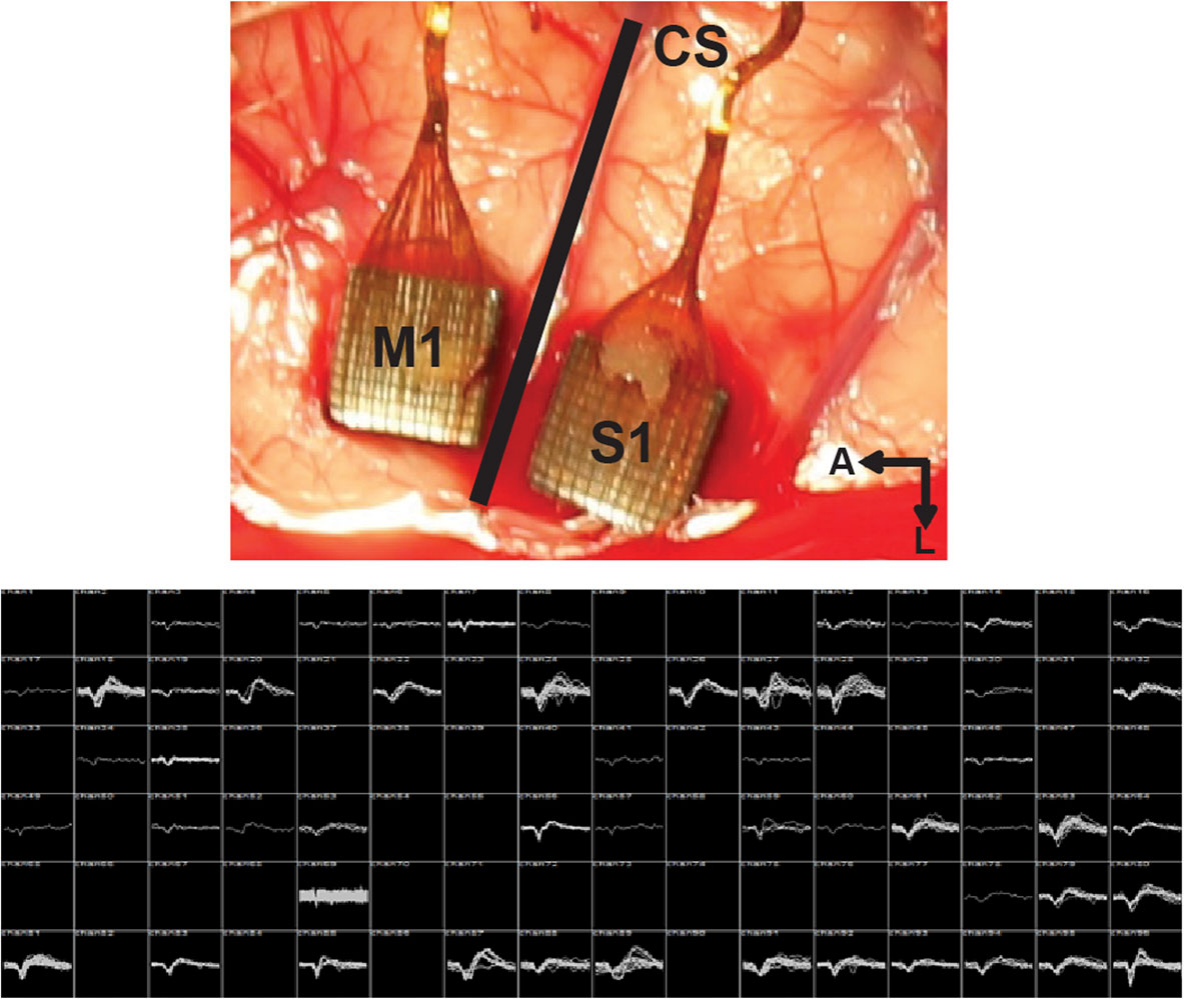
(Top) Surgical photo of Utah arrays implanted in the motor and sensory cortex of a rhesus macaque. A – anterior, L – lateral, CS – central sulcus. (Bottom) Spike panel recorded from the M1 array using a Cerebus Neural Signal Processor (Blackrock Microsystems) illustrating the number of single units and their quality. Data from this monkey was used in later analysis to validate the device offline and in vivo

Two rhesus macaque monkeys were induced under general anesthesia and placed in a stereotactic frame. The craniotomy site was located using the stereotactic frame to estimate the location of the central sulcus. The hand region of primary motor cortex (M1) was approximated by projecting a line from the genu of the arcuate sulcus posteriorly toward the gyrus immediately anterior to the central sulcus. The location of hand in the somatosensory cortex (S1) was approximated as the gyrus immediately posterior to the central sulcus across from the motor hand region. Two 4 mm × 4 mm, 96-channel, intracortical Utah arrays (Blackrock Microsystems) were implanted in motor and sensory hand region as shown in Fig. 5.

Broadband data were recorded at 30 kSps from the arrays using a Cerebus Neural Signal Processor (Blackrock Microsystems). Neural spikes were detected by high-pass filtering the raw data at 250 Hz and thresholding the resulting signal at − 4.5 times the RMS voltage on each channel, similar to other experiments (Gilja et al. 2015).

#### Experimental setup

##### Data playback

**Fig. 6:**
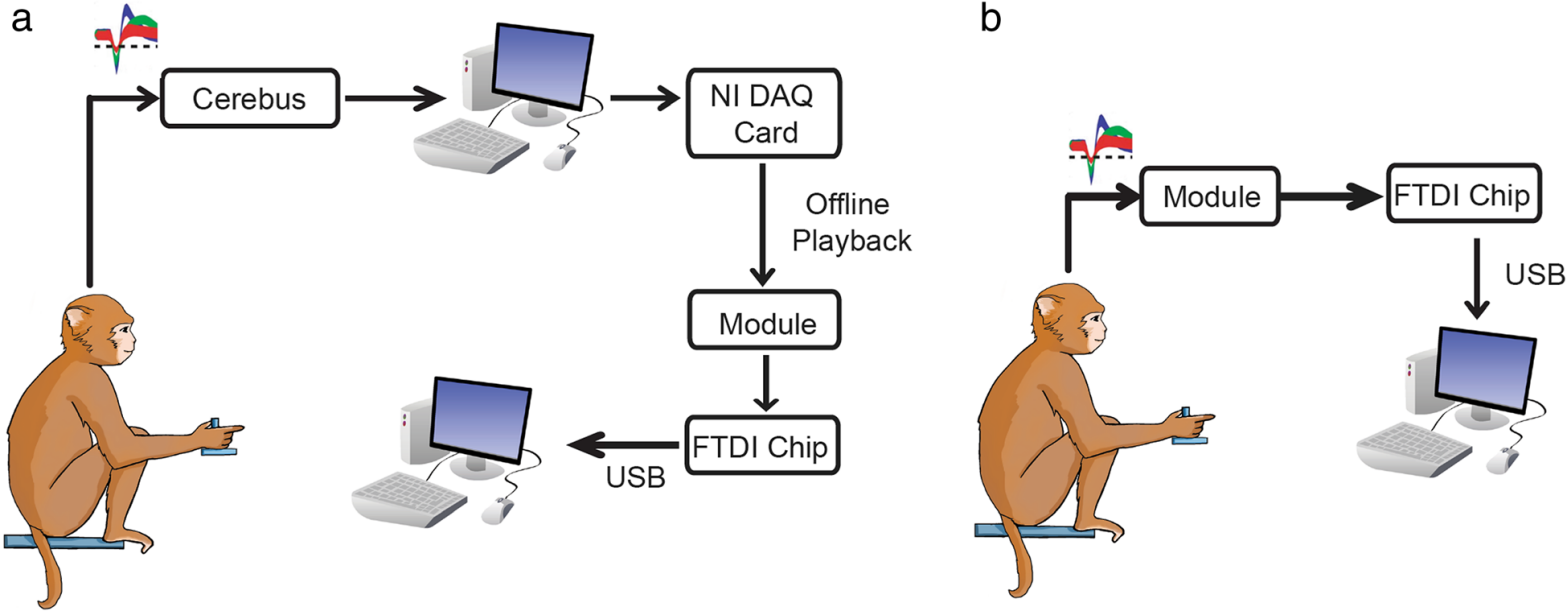
Experimental Setup. **a** While the monkey performs the finger flexion task, broadband neural data is recorded through the Cerebus and saved on a computer. The offline data is later replayed through the module using a National Instruments DAQ card. **b** While the monkey sits still, neural data is recorded through the device and sent to a computer

Validation of the module in both single channel and multichannel modes were performed using pre-recorded data. Broadband data that had been previously recorded through the Cerebus (Blackrock Microsystems) and saved to an external computer were replayed through the module using a National Instruments DAQ card (PCI-6711) and then sent to a computer to view for further analyses, shown in Fig. 6a. The DAQ was a 12-bit, 4 channel, 1 MS/s analog output device. This was used in conjunction with a shielded connector box (NI SCB-68A) to access the channels and connected to the module via Samtec connectors. The output of the DAQ was adjusted via a voltage divider in order to achieve the original signal amplitude and outputted at the original recorded sampling rate at 30 kHz.

Sending known pre-recorded data through the device allowed for verification that the output data was correct. Additionally, whereas the general spike waveform is known and can be visually detected in the signal, the spiking band waveform is not and offline comparison with the exact same data is critical. In practice, the system will not need to send neural data off the device. A motor command will be decoded from the data and sent over the NNP system to be transformed into a stimulation pattern. However, for testing purposes, the recorded data was sent to a computer for analyses and validation of the module. In 2 kSps mode, at the end of each bin period, the module computes the average for each channel. At the completion of either 2 kSps or raw 30 kSps data, the device passes all the recorded data to an external computer through USART, using an FTDI chip as a USB interface for analyses and validation. Neural action potentials are visible when in single channel mode and the spiking band power is visible in multichannel mode and are later compared to PC-processed data.

##### In vivo recordings

Data was also recorded by the module in vivo directly from a monkey. During these recordings, the monkey sat in a primate chair (Crist Instruments) with his head restrained not performing any task. A Cereport breakout connector was used to connect the array pedestal to the module through the Samtec connectors. Neural data was recorded directly through the device and sent to an external computer using an FTDI chip as a USB interface, shown in Fig. 6b.

## Results

### Device validation

Figure 3 shows the final device, 1 cm × 4 cm for each of 3 panels. Currently this prototype does not include the flexible connections between panels, but only 16 lines run between the panels, such that the same layout could be fabricated in a flex design. An example of this flex design is illustrated in Fig. 4. This design was tested extensively on the benchtop and with animals as described below.

#### Pre-recorded data

**Fig. 7:**
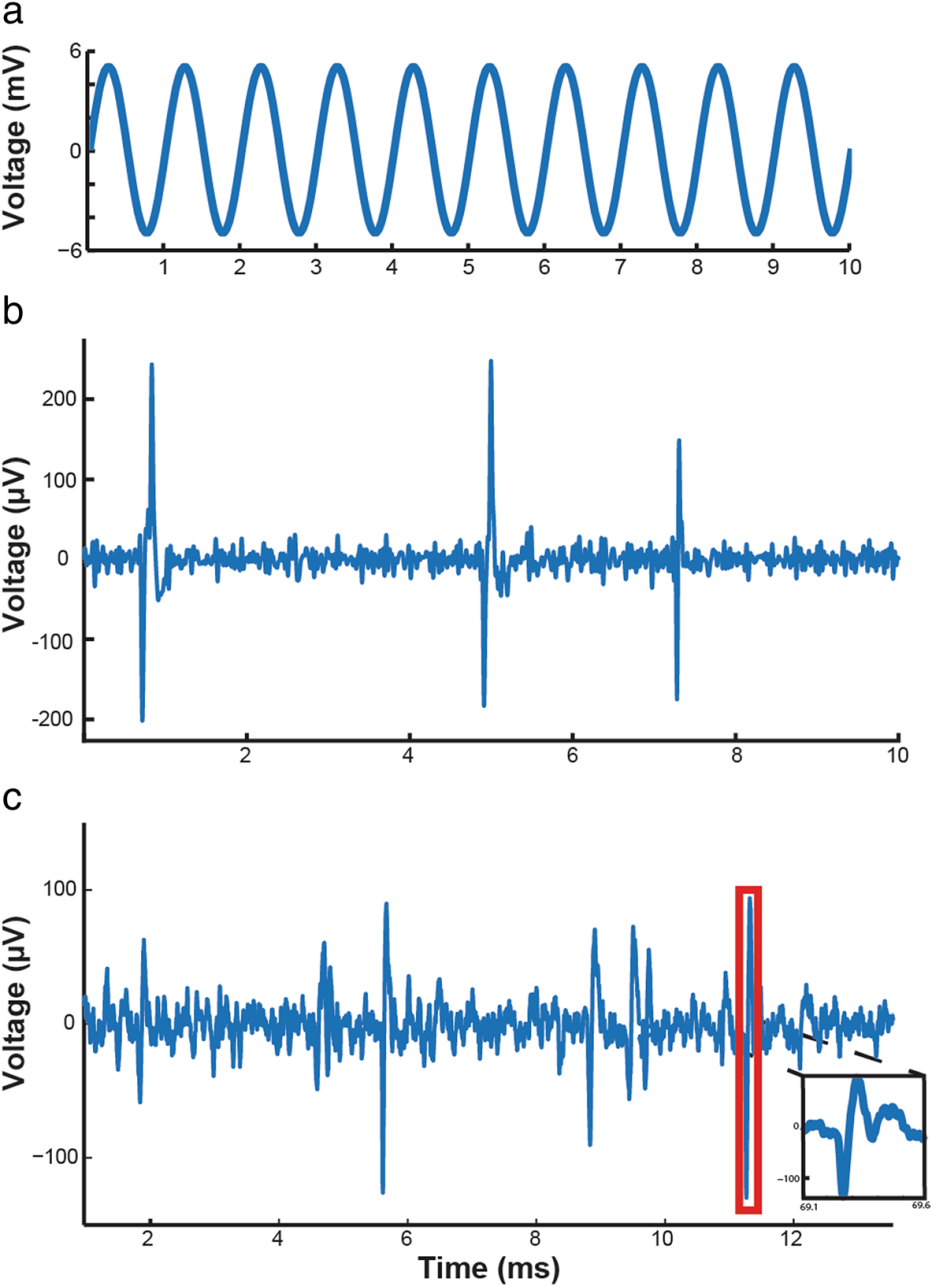
Single channel data recorded through the device, sampled at 32 kSps. **a** 1 kHz sine wave. **b** Simulated neural data from neural signal generator (Blackrock Microsystems). **c** Pre-recorded neural data from rhesus macaque

First, we verified that signals were passing reliably through the Intan amplifiers to the microcontroller. During these tests, the module was powered by a DC regulated power supply at 3.3 V, bypassing the built-in power conditioning circuitry. The amplifiers were configured to filter a passband of 0.1–7500 Hz and were sampled at 32 kSps. These settings enabled testing of the wideband performance of the device. The sampled data was transmitted to an external computer for storage and processing. In single channel mode, signals were introduced and verified at individual inputs on each bioamplifier. Individual channel investigation was necessary, as the system was designed to process data at much lower rates and the microcontroller cannot handle the throughput necessary for all 96 channels at 32 kSps. However, the spiking band of all 96 channels can be processed in multichannel mode at 2ksps. Figure 7 shows the results of testing in single channel mode, where details of the incoming signal can be easily viewed. First, a 1 kHz sine wave was introduced to the amplifier input and the sampled output at 32 kSps, shown in Fig. 7a. The device was next validated with simulated neural signals from a neural signal generator (Blackrock Microsystems). Figure 7b shows an example recording where spikes were clearly visible. Figure 7c shows an example using real, prerecorded neural data at 32 kSps from a rhesus macaque performing finger flexion tasks. All channels looked and performed similarly.

#### In vivo data

**Fig. 8:**
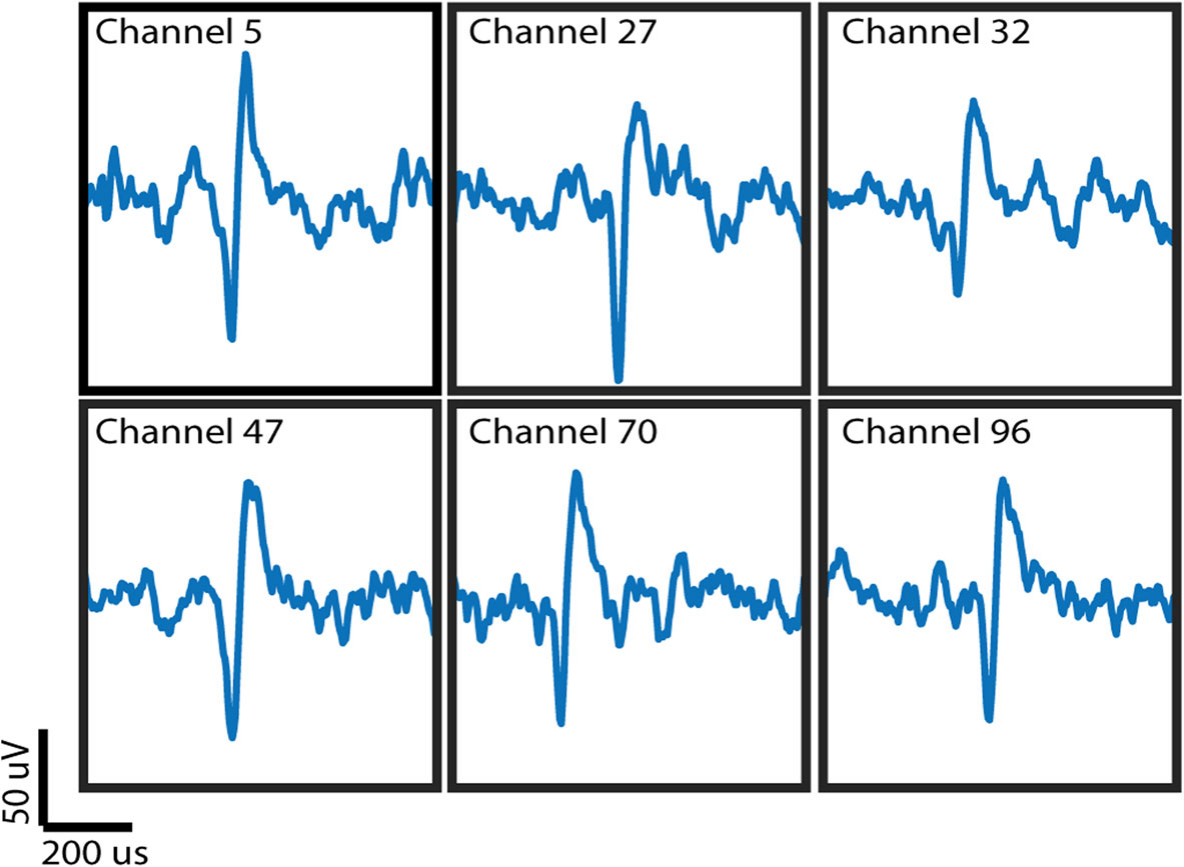
Single units recorded in vivo through the device. Each channel was recorded individually

For consideration as a future clinical system, the device also needs to be able to handle the noise and impedances associated with live recordings. We tested this by recording directly from a Utah array in a nonhuman primate. Figure 8 shows an example recording from six channels showing clear single unit activity, manually picked out. Data recorded through the device had an RMS noise of 3.2 μVRMS which is comparable to that of the Cerebus at 2.1 μVRMS. These channels were recorded separately, as this device is not designed to process 30 kSps data on all 96 channels, as the MCU cannot sample that fast and it would consume too much power for the overall system. However, these results show it is possible to record one channel of broadband data for basic science purposes, or for calibration purposes in a clinical system.

### Spiking band power validation

#### Module outputs spiking band power

**Fig. 9:**
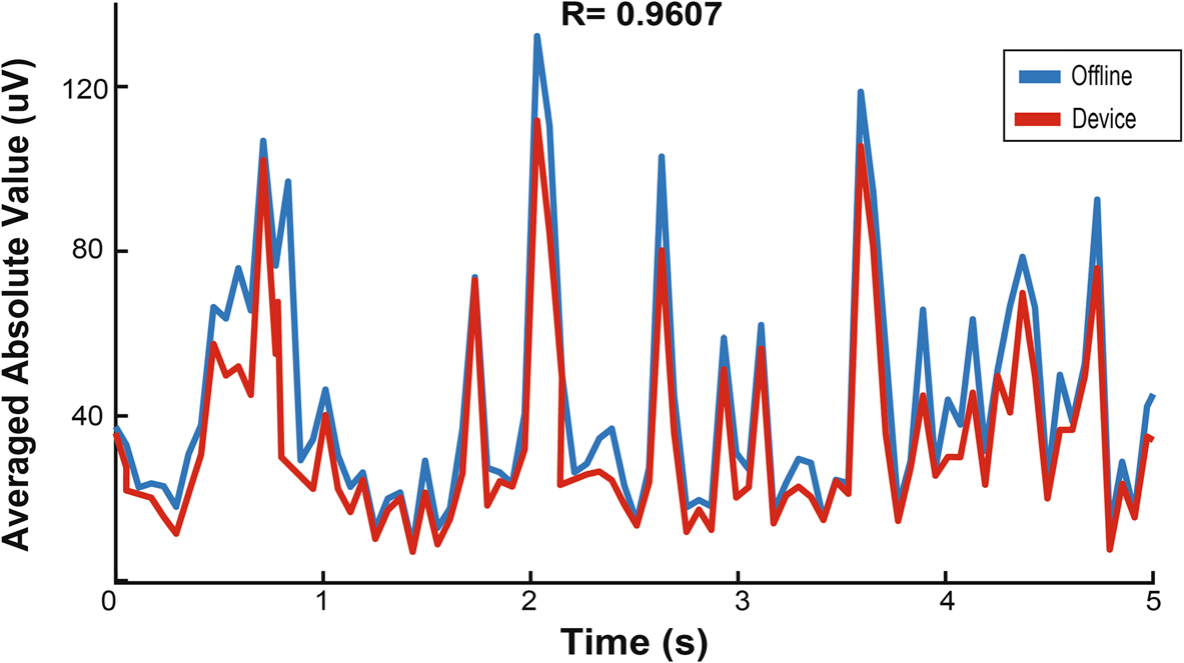
Power calculated from 2 kSps data offline compared to data output from the device on a single channel

Next, we tested the intended usage mode where all 96 channels are recorded at once, using a spiking band filter similar to that used by Stark and Abeles (Stark and Abeles 2007). The device was configured to filter inputs between 300 and 1000 Hz, which we previously showed to enable decoding with 95% of the performance associated with decoding threshold crossing events (Irwin et al. 2016). The microcontroller sampled all 96 channels at 2.17 kSps and transmitted the averaged absolute value of the signal after 128 samples (~ 58 ms bins) to a computer for analysis. This mode was tested with raw pre-recorded neural data from Utah arrays, played back through the device, such that it could be compared in multiple ways. We performed the same processes used on the device offline in Matlab. The same broadband dataset used to playback through the device was filtered between 300 and 1000 Hz with a 2nd order Butterworth filter, downsampled to 2 kSps, and the absolute value was averaged over 58 ms to estimate the mean signal power on each channel. The output from the pre-recorded data played back through the device was compared to the offline processed result. The offline Matlab results and the device output are shown in Fig. 9, where the two signals matched closely, with a Pearson’s correlation coefficient of 0.9607.

#### Comparing spiking band to spiking rate

**Fig. 10:**
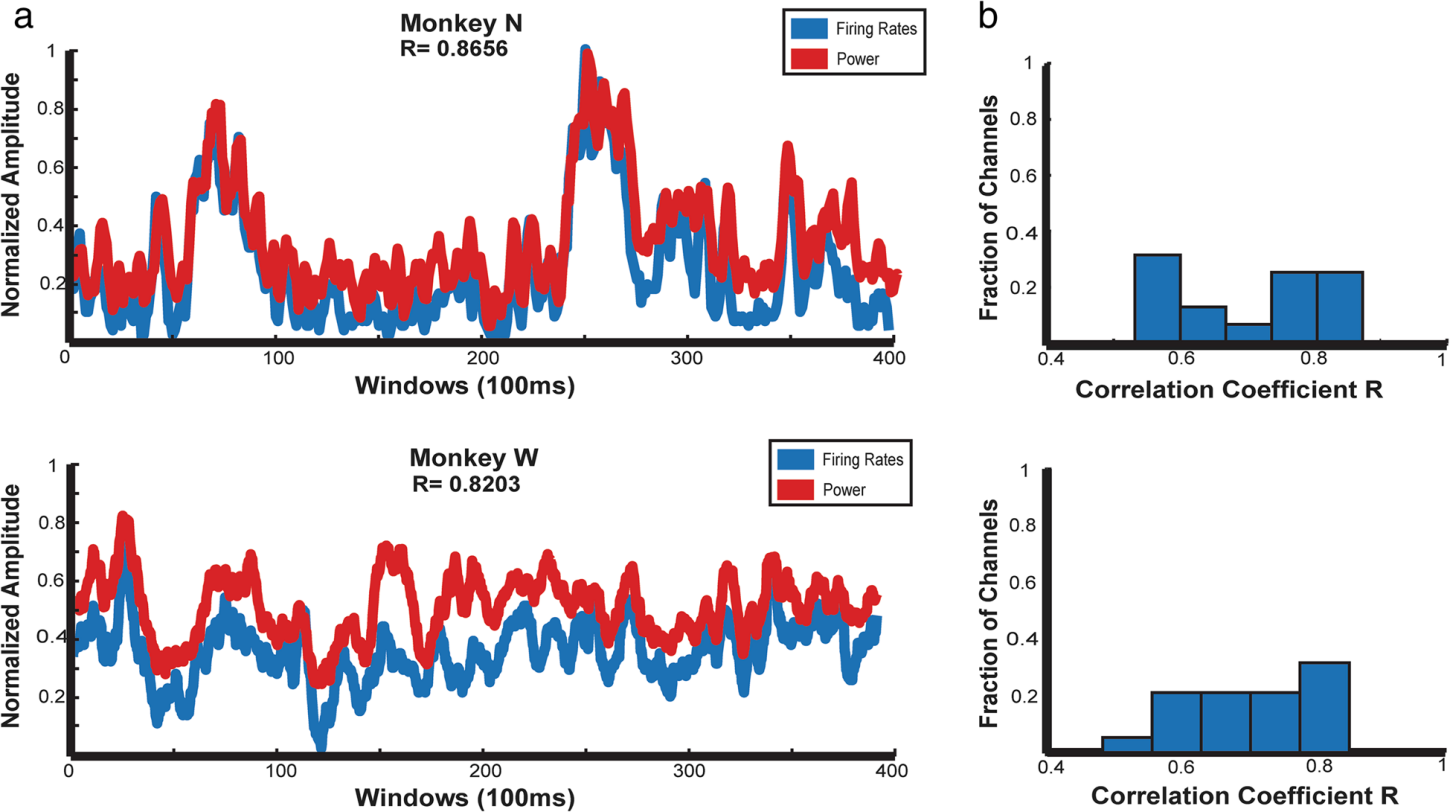
**a** Comparison of mean spiking band power from the device normalized to the maximum power and firing rate calculated offline normalized to the maximum firing rate over 100 ms windows for the best single channel. **b** Histograms of the correlation coefficients for all channels in the dataset

In Irwin et al, we showed that low bandwidth intracortical data could be used to predict a monkey’s continuous finger position. In comparison to decodes using spike counts acquired from high bandwidth data, performance drops by only 4.9% (Irwin et al. 2016). One explanation of this is that signal power within 300–1000 Hz represents the firing rate of neurons on a particular channel, leading to a similar decode performance. To further test whether the spiking band power results from actual spikes, we compared the spiking band power output from the device to the firing rate obtained from thresholding the broadband data, as used in many online BMI experiments. Datasets were chosen for the two monkeys (N, W) on days with similar tasks and performance. Within these datasets, the channels with visualized single unit waveforms were used in the analyses. Using Matlab, the mean firing rate was calculated in 100 ms windows. The same broadband datasets were replayed through our device, which returned the 2 kSps spiking band power, which was then averaged offline in Matlab with the same 100 ms window size. We compared the traces of mean firing rate and mean power over multiple channels and used the correlation coefficient to assess their agreement. This yielded a mean correlation of R = 0.8656 in Monkey N and R = 0.8023 in Monkey W. The high correlation between firing rate and signal power suggests that the spiking band is related to actual spiking events and is not only capturing local field potentials (LFP). The channel with the best performance in each monkey is shown in Fig. 10a. Histograms of per-channel correlations are shown in Fig. 10b. The varying distribution of correlation across the channels and different animals can be attributed to the variance of the amplitude and activity frequency of the neurons during the defined time window.

### Power consumption

In each mode, power was calculated by measuring the total system current through a jumper. Most of the power was consumed by the three Intan RHD2132s and the Atmel AT32UC3C2256C microcontroller. With all power saving techniques in use, system power was measured at 33.6 mW while sampling 96 channels at 2 kSps, configured to filter between 300 and 1000 Hz. As a comparison, the power required to transmit a single channel, filtered between 0.1–7500 Hz at 30 kSps, using our system was 31.2 mW. To transmit a single channel at 2 kSps, only 22.1 mW were needed. The primary single-channel power savings came from the reduced filter bandwidth and sampling rate. While it would not be practical to stream 96 channels at 30 kSps, it is possible to view single unit waveforms one at a time using this approach.

To achieve this lower power consumption and ease the burden on the overall system power management of the NNP, we used several features of the MCU to decrease overall power consumption, summarized in Table 2. First, we enabled the DMA which allowed the device to control sampling from the amplifiers without having to wake up the processor from a low-power sleep mode. The DMA was more efficient at sampling and was necessary to acquire data from all 96 channels at 2 kSps. The core alone could manage a maximum of 1 kHz while at the full clock rate, while the DMA could still exceed the sampling rate even after a major reduction in clock speed. System power was measured at 45.3 mW while transmitting all 96 channels at 2 kSps using the DMA. Next, we disabled all unused peripherals on the MCU. Using the DMA and turning off unused peripherals decreased power consumption by 11% to 40.0 mW. Finally, the processor core was configured to spend most of its time in IDLE sleep mode. Since the DMA automates sampling, the MCU core only needs to wake up every 64 ms to bin the data. With all these power saving techniques enabled, system power was measured at 33.6 mW, a 16% reduction from when the CPU is fully awake, and a 25% reduction overall.

**Table 2:** Power Saving Techniques

No. Channels	Power Mode	System Power
1	30ksps	31.2 mW
1	2ksps	22.1 mW
96	2ksps DMA sampling + CPU awake	45.3 mW
96	2ksps DMA sampling + Disabled Peripherals + CPU awake	40.0 mW
96	2ksps DMA sampling + Disabled Peripherals + CPU asleep	33.6 mW

In addition to the techniques discussed above, reducing the number of channels transmitted could also reduce the amount of power consumed by the device. Selectively using only the well-tuned channels has been shown to positively contribute to the decode performance (Wahnoun et al. 2006). This concept of channel masking allows us to record from only a subset of channels and retain the decode performance. We simulated this by recording from only a portion of the channels evenly distributed across all three amplifiers. Transmitting all 96 channels in the lowest power mode required 33.6 mW. Disabling roughly a third of the channels to transmit only 63 channels consumed 31.4 mW. Disabling approximately two thirds of the channels allowed a 50% reduction to the system clock speed, where transmitting 33 channels of data required 26.7 mW.

Additionally, for completeness, we tested functionality of the device’s CAN network interface and power conditioning circuitry by connecting it exclusively to an NNP power module and validated that the devices could communicate. System power of the neural recording module was measured at 43.6 mA, while being powered by the NNP power module at 3.98 V, and recording and binning all 96 channels, using the DMA as described above.

## Discussion

We have designed a 96-channel neural recording module prototype that can be used with the NNP system. The neural recording module was designed using entirely off-the-shelf components and all active circuitry fit within 1 cm × 4 cm panels. While the current NNP sensor modules have a 1 cm × 3 cm hermetically sealed enclosure, increasing the length by 1 cm is not outside of the capabilities of the manufacturer and does not compromise the design. Our device extracts signal power in a narrow frequency band, which allows us to sample at a low rate of 2 kSps. We demonstrated the neural recording module consumed 33.6 mW when transmitting all 96 channels at 2 kSps using all power saving techniques. We have not yet implemented decoding and full communication over the CAN network and expect these functions to draw some additional power. However, we have tested the functionality of the CAN network while the device was being powered exclusively by the NNP power module and found our device’s power consumption to be similar to that of existing NNP permitting normal function of other modules (power, actuator, and sensor). Most demonstrations of wireless neural recording devices have been done using > 50 Mbps of digitized voltage traces, which is far beyond the specifications of implantable devices. The ability to sample at a lower rate tremendously decreases the size of the data and will be key in communication with the NNP network, which has a bandwidth of 100 kbps. Our current design uses three 8 mm × 8 mm 32-channel Intan bioamplifiers, which is a limiting factor in PCB length and power consumption of the module. However, the BGA packaging of the next generation 9 mm × 7 mm 64-channel Intan bioamplifier offers the possibility of improving our current design by decreasing the number of chips from 3 to 2. This will dramatically reduce the size of our board without any major design changes, bringing us closer to the 1 cm × 3 cm package of the existing NNP modules. Each individual bioamplifier consumes some baseline power, so the reduction in the number of Intan bioamplifiers required will eliminate a portion of this power consumed and enable significant power savings. In addition, if the design option were available to reduce the 3.3 V supply voltage required for the Intan bioamplifiers, that would lead to further reduction of the overall power consumption of the device.

Additionally, channel masking provides opportunities to save even more power. Wahnoun et al. found that less than 70% of recorded neurons are well tuned to movement direction and that some of these neurons actually decrease decoding performance. Selecting the best 20 neurons to control their neuroprosthetic system performed better than using all neurons (Wahnoun et al. 2006). Reducing the number of channels transmitted will reduce the computational load and result in a decrease in overall power consumption and prolonged battery life.

While it enables dramatic power savings, there are still open questions about exactly where the “spiking-band” signal is coming from. This is important in order to understand how far the approach can go. Is it a local signal directly reflective of spikes? Or is it broader LFP like electrocorticography (ECoG), which can also contain some movement information (Chestek et al. 2011; Flint et al. 2016)? We have previously demonstrated that we can drop the upper frequency of the spiking band filter from 6 kHz (Stark and Abeles 2007) to 1 kHz with only a 4.9% decrease in performance (Irwin et al. 2016). Here, we have shown that the power in the spiking band is highly correlated with the firing rate of threshold crossing spiking rate of the input high bandwidth data. This suggests that spiking band power may be most reflective of the spikes themselves. This is not surprising since action potentials have a 1–1.5 ms sinusoidal-like waveform, and we filtered between 300 and 1000 Hz. Spike amplitude also falls off quickly, theoretically as the reciprocal of the distance from the electrode (Holt and Koch 1999; Moffitt and McIntyre 2005), which makes it possible that the spiking band signal is fairly local. Based on the frequency components it is likely that the spiking band contains both localized spikes and broader LFP. This may be a benefit to the traditional use of thresholded spikes because the spiking band may be used to still interpret useful data from channels that over time no longer have good quality spikes.

Custom ASIC designs have been presented to be smaller in size and consume less power, however, the noise, impedance, and large transient voltages that are present in live data within real world settings make it difficult to also achieve signal integrity and stability under these conditions. With academic bioamplifiers progressing to a more commercial platform, we can record live stable signals under very realistic parameters using off the shelf components, as seen with this device. Overall, using the signal processing techniques described here, custom ASIC-free designs may have crossed the threshold of viability for multi-channel neural recording.

At the moment, percutaneous wires and power are arguably the most limiting factors in translating BMI systems to clinical use. The best devices run for only a few hours on a battery or rely on wearable components (Borton et al. 2013). So long as this is true, spiking band devices may offer the fastest path to clinical viability. In the future, should the number of electrodes become more of a limitation than the power, it may make more sense to extract the maximum amount of information from each electrode by extracting single unit timing. Currently, however, several hundred electrodes can be implanted percutaneously in humans (Aflalo et al. 2015; Collinger et al. 2013; Gilja et al. 2015; Hochberg et al. 2012), without being able to process even 100 of those channels with implantable electronics.

## Conclusion

This device was designed to acquire data at configurable bandwidths, sampling rates, and channel counts using the Intan bioamplifiers which could allow it to be used for applications outside of Utah array recording. The freedom to choose the filter parameters supports a variety of neural signal modalities, such as ECoG and EMG. While there are many interesting implantable devices in development for EMG (Baker et al. 2010; Troyk et al. 2007) as well as wireless ECoG for epilepsy (Mestais et al. 2015; Vansteensel et al. 2010), these devices would serve relatively small markets by themselves. However, the concept behind the NNP is that existing modules can be applied to multiple applications and markets, as the stimulation and power modules can be used for a brain-controlled FES device theoretically proposed in this work. The specifications in this paper, representing the first attempt to combine designs from different groups, can be used as a guide to develop further application specific modules. While representing a departure from traditional medical devices, the reuse of components and circuitry from the existing NNP platform may dramatically accelerate the overall process to a clinically viable system within the scope of bioelectronic medicine.

## Abbreviations

ADC: Analog to digital converter
BMI: Brain machine interface
CAN: Controller area network
DAQ: Data acquisition
DMA: Direct memory access
ECoG: Electrocorticography
EMG: Electromyography
FES: Functional electrical stimulation
MCU: Microcontroller
NNP: Networked Neural Prosthesis
PCB: Printed circuit board layout
RMS: Root mean square
SCI: Spinal cord injury
SPI: Serial peripheral interface
